# Supervising community health workers in low-income countries – a review of impact and implementation issues

**DOI:** 10.3402/gha.v7.24085

**Published:** 2014-05-08

**Authors:** Zelee Hill, Mari Dumbaugh, Lorna Benton, Karin Källander, Daniel Strachan, Augustinus ten Asbroek, James Tibenderana, Betty Kirkwood, Sylvia Meek

**Affiliations:** 1 Institute of Global Health, University College London, London, UK; 2 Malaria Consortium, London, UK; 3 Department of Population Health, London School of Hygiene and Tropical Medicine, London, UK

**Keywords:** supervision, community health worker, lay health worker, health worker, low-income country, developing country, ICCM

## Abstract

**Background:**

Community health workers (CHWs) are an increasingly important component of health systems and programs. Despite the recognized role of supervision in ensuring CHWs are effective, supervision is often weak and under-supported. Little is known about what constitutes adequate supervision and how different supervision strategies influence performance, motivation, and retention.

**Objective:**

To determine the impact of supervision strategies used in low- and middle-income countries and discuss implementation and feasibility issues with a focus on CHWs.

**Design:**

A search of peer-reviewed, English language articles evaluating health provider supervision strategies was conducted through November 2013. Included articles evaluated the impact of supervision in low- or middle-income countries using a controlled, pre-/post- or observational design. Implementation and feasibility literature included both peer-reviewed and gray literature.

**Results:**

A total of 22 impact papers were identified. Papers were from a range of low- and middle-income countries addressing the supervision of a variety of health care providers. We classified interventions as testing supervision frequency, the supportive/facilitative supervision package, supervision mode (peer, group, and community), tools (self-assessment and checklists), focus (quality assurance/problem solving), and training. Outcomes included coverage, performance, and perception of quality but were not uniform across studies. Evidence suggests that improving supervision quality has a greater impact than increasing frequency of supervision alone. Supportive supervision packages, community monitoring, and quality improvement/problem-solving approaches show the most promise; however, evaluation of all strategies was weak.

**Conclusion:**

Few supervision strategies have been rigorously tested and data on CHW supervision is particularly sparse. This review highlights the diversity of supervision approaches that policy makers have to choose from and, while choices should be context specific, our findings suggest that high-quality supervision that focuses on supportive approaches, community monitoring, and/or quality assurance/problem solving may be most effective.

Community health workers (CHWs) are an increasingly important component of many health systems and programs and deliver a wide range of health interventions (1) including the management of sick children through initiatives such as Integrated Community Case Management. Adequate supervision is considered key to ensure that CHWs perform well, are motivated, and have well-defined roles in the community and in relation to the health system (2–6). Yet, despite its recognized importance, supervision is often lacking in quality if it is present at all.

Although supervision has a long history, with paradigm shifts well documented and discussed, surprisingly little is known about what constitutes adequate supervision and how different supervision strategies influence performance. Effective and regular supervision could potentially help meet the challenges unique to CHWs, especially in the context of task-shifting initiatives that transfer tasks from formal health workers to CHWs (7–9). Supervision could help focus CHW efforts and identify and correct poor practices (10). Exploratory studies have consistently identified quality supervision as a positive contributor to CHWs’ job motivation, retention, and satisfaction (5, 11, 12); however, if done poorly, supervision can also contribute to CHW dissatisfaction (10). Finally, supervision by formal health workers gives CHWs a sense of legitimacy in the eyes of other health workers, the communities served by CHWs, and CHWs themselves (13).

Formal health worker supervision can be dated back to the early 1900s, when it was conceived as an organizational and management process (14). In developing countries, the need for supervision was highlighted during the primary health care movement where remote workers were perceived as needing supervision to link them to the health system and to supplement their training. By the start of the decade, it was recognized that traditional supervision, emphasizing inspection and control of health workers, was not working and there was a move toward supportive or facilitative supervision focusing on providers’ needs and collaborative problem solving (15, 16). This move was influenced by the emergence of models of clinical supervision from high-income countries (17–25), which take varied theoretical and practical stances to supervision but have been criticized for failing to demonstrate how they can be practically applied (25, 26).

Supportive supervision is considered best practice and usually contains elements of record reviews, observations, performance monitoring, constructive feedback, provider participation, problem solving, and focused education. In practice, supportive supervision strategies vary greatly in approach, content, and tools (27), and there is little empirical evidence to help those implementing CHW programs design effective supervision systems that address the unique qualities that characterize CHW's roles in the community and relationship to the health system.

Despite the recognized role that supportive supervision can play in performance and motivation, numerous studies from a range of countries and programs have found that supervision often has low coverage; low administrative focus; is irregular, unsupportive, and demotivating; and lacks adequate training for supervisors and problem solving or feedback mechanisms for providers (11, 16, 27–32). For example, in a time-use study in Ghana, only 15% of workers reported feeling supported by their supervisor (33). These shortcomings are caused by a range of issues including geographical, financial, human resource, and transport problems; lack of tools, coordination, skills, and training; competing responsibilities; poor motivation; and hierarchical systems based on a culture of top-down management and control rather than collaboration (16, 28, 30, 34–56).

Given the increasing use of CHWs to deliver health services and the documented role quality supervision can play in maintaining health worker and CHW performance (57, 58) and motivation (5, 11, 59–61), identifying effective supervision strategies and addressing implementation issues could help improve CHW supervision and motivation, performance, and outcomes. This paper is a literature review of different supervision strategies for a range of health care providers in low- and middle-income countries with a focus on the implications for CHWs. The review was conducted as part of the inSCALE study in order to identify potential approaches to improve the motivation, retention, and performance of CHWs to be tested in a randomized control trial (62).

## Methods

We conducted a review of literature evaluating the impact of supervision interventions and/or identifying implementation issues. For the impact component, we located English language articles published in peer-reviewed journals up until November 2013 through a PubMed and Web of Science search using combinations of the following search terms: Supervis*, developing countries, low-income countries, health worker, community health worker, community-based agent, lay health worker, and community volunteer. A hand search of reference lists, relevant web sites and bibliographies was also conducted. Inclusion criteria were that the article evaluated the impact of supervision in a low- or middle-income country using a controlled, pre-/post- or observational design. Review articles and commentaries were excluded as were articles describing but not testing strategies. The inclusion criteria for articles exploring implementation issues were broader and included gray literature such as program reports and qualitative papers that described implementation and feasibility issues; these were identified through web searches. Articles that described general barriers to supervision were excluded as the review focused on barriers to specific supervision strategies; guidelines for supervision were also excluded. After detailed reading of each paper, we classified each intervention based on the strategy being tested and synthesized and collated the findings for each strategy. The quality of the paper was judged based on the potential for selection bias (whether a control group was used; allocation to this group and similarity of intervention and control groups at baseline; whether assessors were blinded; and levels of loss to follow-up) and reporting bias (selective reporting of outcomes).

Implementers and those with program experience are a valuable source of information on the effectiveness and feasibility of supervision strategies, and their views offer a useful addition to published reports, key stakeholders were interviewed on their perspectives and experiences with supervision with the results reported elsewhere (5).

## Results

We identified 22 papers, published between 1984 and 2013, relating to a range of low- and middle-income countries addressing the impact of supervision of a range of health care providers and using various evaluation methods (see Table 1). We classified interventions as testing supervision frequency, the supportive/facilitative supervision package, supervision mode (peer, group, and community), tools (self-assessment and checklists), focus (quality assurance/problem solving), and training. Below we report the findings for each type of intervention summarizing the impact and implementation issues.

**Table 1:** Summary of impact studies

	Number of studies
Study design	
Randomized control trial	4
Non-randomized control trial	8
Before and after	4
Multivariate/observational	6
Supervisee	
Health workers	14
Doctors	1
Immunization providers	2
Family planning providers	3
Community health workers	2
Supervision strategy*	
Frequency	7
Supportive packages	2
Mode	6
Tools	6
Focus	4
Training	2

* Five studies were classed in more than one category.

Overall, the quality of the impact studies was low with only one study classed as high quality (63). The other studies had a combination of small sample sizes, the potential for selection bias, inadequate blinding, high loss to follow-up, and short follow-up periods. Outcomes included coverage, worker performance, and perceptions of quality of care but were not uniform across studies making comparisons and synthesis difficult.

Papers from the early- to mid-2000s tended to focus on supervision frequency, supervisor training, and peer or group supervision while later papers tended to focus on supportive supervision, quality assurance/problem solving, and community supervision. Papers focusing on self-assessment spanned time periods. This change in paper focus reflects the move away from supervision being seen as a management tool and toward supervision being seen as addressing providers’ needs.

### Supervision frequency

Increasing the frequency of supervision is a goal of many programs. However, evidence suggests that increasing frequency alone does not necessarily lead to increased effectiveness. In the only randomized trial we located, exploring the impact of supervision frequency, family planning supervisory visits in Brazil were reduced from monthly to quarterly with no detrimental effect on the number of new clients, revisits, or distributor turnover (44). With an average of 6 min per supervisory visit spent discussing performance compared to 59 min spent collecting service statistics, the authors hypothesize that supervision quality was poor and therefore reduced frequency did not affect worker performance (44). Five multivariate analyses explored the association between supervision frequency and health worker (64–66) or CHW (67, 68) performance in low-income countries; only two found a positive association (64, 68). There was no association between midwife performance scores and supervision frequency in the control group of a study in the Philippines but there was a dose response in the intervention group with improved supervision suggesting that increased frequency only improves performance if quality supervision is in place (37). Similarly, a time-use study in Ghana reported that recently supervised health workers who did not feel supported by their supervisors were no more productive than unsupervised workers, while recently supervised workers who felt supported were much more productive than other workers (OR 2.37, *p*<0.01) (33). Despite methodological limitations, these studies suggest that the quality of supervision could play an equally if not more important role than frequency in intervention efficacy.

### Supportive/facilitative supervision package

Although considered best practice, we located only two peer-reviewed evaluations of supportive supervision. Both suggest that supportive supervision increases worker performance and quality of care. A randomized control trial from Benin of health workers trained in Integrated Management of Childhood Illness (IMCI) conducted a per-protocol analysis using a pre-/post-design with non-randomized controls due to slow IMCI implementation (54, 69). Three years after implementation, they found a 27% point difference in children receiving recommended care in intervention compared to control areas with routine supervision and a smaller impact on the proportion of IMCI tasks performed (54, 69). In India, the effect of supportive supervision on immunization providers was measured using a before and after design. Performance was measured using a checklist, administered by the supervisor, covering management, practices, and supplies with a 36% (*p*<0.01) increase in worker performance scores 18 months after implementation (70).

A review of supportive supervision from several, mostly small-scale NGO projects, shows that supportive supervision is feasible but requires motivation, leadership, time, investment, and a shift in behaviors and attitudes (51). We identified several challenges to implementation including difficulties maintaining supervision coverage and motivation (54, 71), the need for initial intensive external assistance in initiating supervision (70), the time intensiveness of the process (70, 72), difficulties organizing lengthy supervisory visits, unproductive observations due to slow client flow (72), and supervisors failing to provide timely or any feedback post-visit (73). Ensuring that the supportive supervision process is simple and easy to implement has been recommended (72).

### Peer supervision

Peer supervision can take many forms including observation and feedback, stronger peers supporting weaker performers and group meetings to discuss and solve problems. It is considered a beneficial strategy as peers can empathize with each other outside of a hierarchical setting (5) and may be an alternative strategy where traditional supervision is too costly (74). In some CHW programs, good performers have been promoted to supervisors providing a career pathway and source of job motivation for CHWs (75).

No studies were identified evaluating peer supervision alone; however, two non-randomized control trials, one in Indonesia (74) and one in Mali (76), examined the impact of peer supervision combined with self-assessment. In addition, in Indonesia, a non-randomized control study evaluated peer training (77). All of these studies showed some positive impacts: In the Indonesian trial, arms of weekly self-assessment alone and in combination with peer group meetings were compared to standard supervision of family planning providers. Communication indicators for providers and clients in the self-assessment alone arm increased by 5 and 3%, respectively, while self-assessment combined with peer group meetings resulted in a 9% increase in communication indicators for providers and 5% increase for clients; client ratings only increased in the self-assessment group (74). Both peer observation with weekly self-assessment in Mali (76) and peer training in Indonesia (77) resulted in some improved health worker performance. Health worker management of fever in Mali was 10% higher in the intervention group compared to control groups, although no impact was seen on counseling or quality standards compliance (76). Indonesian health workers with low vaccine coverage spent 1–2 weeks training with a stronger performing peer and achieved a net difference in coverage of 39% between intervention and controls, although some of this difference could be due to improved reporting (77).

Although none of the studies measured the quality of peer discussions or feedback, peer group meetings were highly attended in Indonesia (74) and peer mentoring was liked by both participants and managers (77). Peer training/mentorship shows the most promise but requires particular facilitating capacities such as a system to identify weak and strong performing providers. Despite its potential benefits and popularity, peer supervision may not be best practice in all settings as it could create tension between workers (78), workers may not challenge each other or be able to recognize weaknesses that they share with colleagues, and high workloads (26) or staff turnover (74) could reduce feasibility.

### Group supervision

Group supervision involves multiple providers coming together for a facilitated meeting with a supervisor, usually to collect data, discuss problems, and receive training. This strategy allows supervisors to cover a larger geographic area at a lower cost (79), thereby using time and resources more efficiently. Efficient use of time and increased interactions were specifically cited as benefits to the group approach by managers in a non-randomized control trial in Guatemala, the only study located exploring group supervision (38). By replacing one of two annual individual family planning supervision visits with a group meeting focused on problem solving, routine activities and training, the intervention group achieved 86% supervision coverage versus 60% coverage in the group receiving standard supervision (38). There were non-statistically significant differences in family planning coverage as measured by a couple of years of protection and in some client and provider satisfaction scores (38). This study suggests that group supervision is at least as effective as standard supervision and may be more feasible in some settings.

### Community supervision

Community supervision is based on the premise that communities can hold providers accountable if they have relevant information about the delivery of services and patient rights (63). In a rigorous randomized control trial, Ugandan communities held community meetings with health workers to develop an action and monitoring plan based on health facility performance data (63). Both intervention and control areas continued to receive routine supervision. Quality and utilization of primary health care was higher in the intervention areas with a significant difference in the weight of infants and a 33% reduction in under-5-mortality (63), yet cost and technical skills required for the survey may reduce the feasibility of replicating the intervention in some settings. In other settings, feasibility may be reduced as program observations suggest that community participation can be passive (80). Though methodologically weak, a non-randomized control trial of primary health care units in Thailand found that involving community leaders, in addition to re-training supervisors in identifying and solving problems, had a modest impact on client satisfaction and perceived quality compared to a control group that received training only (81). Community leaders and supervisors received the intervention well despite the fact that they thought it added to the workload of supervisors (81).

### Self-assessment

During self-assessments, providers complete a knowledge test, quality improvement tool or checklist, in the absence of a supervisor, to identify strengths and weaknesses in specific areas. Self-assessments are usually followed by guidance for how to improve practices. Through self-assessment, providers may learn from their experiences, function more efficiently, strengthen their commitment to perform and enhance supervisory visits by reflecting on their performance in advance (78, 82).

We found four non-randomized trials using self-assessment to improve the performance of a range of providers including quality of care of private midwives in Uganda (82), patient–doctor communication with medical students in Mexico (83), health worker compliance with standards of fever care in Mali (76), and family planning counseling in Indonesia (74). In most of these studies, standard supervision continued in all groups (74, 83); however, in the Ugandan study, there were three arms: self-assessment, self-assessment with supervisors trained in problem solving, and a control group with standard supervision. In addition, one pre- and post-study without controls was located using self-assessment to evaluate rural health workers’ performance in mosquito control intervention in Iran (84).

Some studies combined self-assessment with other strategies such as peer supervision (74, 76), quality improvement training, supportive supervision packages, service statistic monitoring, and action plans (82). Self-assessment frequency varied by intervention occurring weekly (74, 76, 83), monthly (76), quarterly (82, 84), or at the provider's discretion (82). All studies reported at least some positive results including modest improvements in communication and interpersonal skills (74, 83), better quality of care/services (76, 82, 84), higher client satisfaction (74), and improved infrastructure, business and management practices (82). The Ugandan study only found improvements in structural and process attributes in the group where supervisors had been trained to problem solve (82). In both studies that reported separate results for use of self-assessment alone and self-assessment with another strategy, the combined interventions showed greater improvements in outcomes over both control groups and use of self-assessment alone (74, 82).

Feasibility issues with self-assessment include finding time and recalling consultations to complete forms, fatigue with repeatedly used forms, staff turnover, initial embarrassment and problems with equipment maintenance when conducting self-assessment by reviewing audio-tapes (74, 76, 83). The effectiveness of self-assessments used in combination with other strategies may rely on the implementation of a package of interventions, and some problems identified by self-assessment may only be solved if efforts are made to increase problem solving abilities (83). The interventions all followed training which may have motivated the providers to complete the self-assessment. There is some evidence that self-assessment requires particular skills and low-performing providers may be less able to assess themselves accurately (78) with the Indonesian study finding a greater impact among experienced providers (74).

### Checklists

Checklists are usually used as part of supportive supervisory packages as they are a way of structuring supervisory visits (51). Several interventions integrated checklists into multi-faceted approaches but only one low-income country study evaluated checklists as a stand-alone tool. In a non-randomized controlled trial in the Philippines, supervisors of midwives were trained for 1 day on the use of a checklist evaluating midwife performance and on giving feedback. Midwives were evaluated on clinical performance, clinic records, and knowledge questions. While control clinics receiving routine supervision increased performance scores by 5 points, intervention clinics increased by 11 points (*p*=0.003) (37).

Both midwives and supervisors in the Philippines accepted the checklist, seeing it as an objective, clear, and concise supervision method (37). The structured nature of checklists appeals to supervisors (38), but checklists can also be time consuming and difficult to implement in busy settings (85) or, if they rely on observations, in settings with low foot traffic (86). Lengthy checklists are not liked (87) and may actually hinder supervision by causing fatigue and automatic use (51).

### Quality assurance and problem solving

Several supervision interventions included a problem-solving component but few reported problem solving as the main intervention or used formal quality assurance tools. One quality assurance intervention in Nigeria did evaluate quality of care with a pre-/post-test targeting the competency of supervisors and the efficacy of the health information system leading to large improvements in the management of simulated diarrhea cases including disease classification and treatment (86).

Several interventions included problem solving and/or quality of care as part of multi-faceted interventions including other components such as self-assessment (82), checklist use (38, 85), group supervision (38), and two-tiered supervision (85). All had some positive impacts, for example, in a study in Guatemala, also described in the group supervision section, family planning providers and supervisors used a checklist to identify and prioritize problems 80% of which were solved (38). A two-tiered supervisory model was used in India where central supervisors visited 10% of clinics quarterly to implement a quality monitoring tool and made recommendations for improvement to be followed up by the state supervisors who were meant to make regular visits (85). Five performance areas of clinical services were evaluated using a pre-/post-design and all improved with the largest increase in coverage of services (85).

While supervisors and providers appear to appreciate problem solving and participatory strategies, these approaches could require additional support (74) as change may conflict with organizational culture, feeling threatened (51), or require external supervisors at least initially (83). For example, in India, supervisors did not like participatory problem solving as they preferred the status of their position in a hierarchical system (51). In terms of formal quality assurance tools, supervisors can understand and use tools such as flow diagrams (86), brain storming for prioritization, and matrix analysis for selection of solutions (87); however, a single training on quality improvement may not be sufficient to garner change (29). Less formal tools such as self-assessments with action plans may be feasible solutions for private providers in remote locations or small practices (82).

### Supervisor training

Almost all of the studies reported in this paper involved training or re-training supervisors but only one non-randomized control study aimed to test specific training models. Supervisors of nurses in South Africa received a one-time training session in one of the two models: the Modified Matrix model focused on the supervisor–supervisee relationship, the institutional and client environment, and the tasks and functions of the supervisor and supervisee, while the Centre for Health and Social Studies model focused on training supervisors in understanding and practicing the principles of primary health care and continuous quality improvement. Neither intervention had significant impact on outcomes, including job satisfaction, patient satisfaction, or quality of hypertension or diabetes care (29); however, the impact of these models may have been limited by not taking a more comprehensive approach. In Zimbabwe, pharmacists and pharmacy technicians without supervision experience were trained to supervise primary health care workers in a three-arm randomized control trial comparing interventions to improve stock management and adherence to treatment guidelines to each other and to a control (88). A comprehensive 2-week supervisor training covered a range of topics including supervision skills, use of checklists, report writing, interpretation of data, and review of stock management and standard treatment guidelines (88). Two supervisor visits to each randomly assigned intervention facility focused on addressing either stock management or treatment guidelines and resulted in statistically significant improvements of overall indicators in both intervention groups as well as improvement in some indicators from the comparator group (88).

## Discussion

Through this literature review, we have described different strategies being used to supervise health providers. Despite an increasing interest in supervision, only one study was published post-2010, and most studies were poor quality and were pilot or small scale. What works and is feasible in small-scale studies may not be directly transferable to scale settings and research at scale is needed. Data on CHW supervision is particularly sparse with only two observational studies focusing on them. Some promising supervision strategies, such as mhealth, targeted supervision, and increasing supervisors’ autonomy, are yet to be evaluated. Our main finding is the lack of rigorously tested strategies and the need for further research, focusing in particular on CHW supervision, implementing supervision at scale and untested strategies.

Classification and synthesis of studies was challenging as intervention components and implementation processes were not always clearly described. Many interventions involved more than one approach and, while combining multiple supervision strategies has been cited as most effective (88), this makes providing data to planners and implementers trying to choose strategies to fit particular needs and contexts difficult. Individual components of interventions evaluated as a package may not have an impact if implemented alone, but it could be difficult to replicate exact packages of interventions in resource or logistically challenging settings. In future, authors should attempt to describe intervention components in more detail for accurate replication. Realist evaluations may provide more useful data for policy makers than randomized control trials as they would allow for a greater understanding of the importance of context.

Despite the poor quality of studies, this review demonstrates the diversity of supervision approaches being implemented in different settings. There is evidence to suggest that improving supervision quality has a greater impact than increasing frequency of supervision alone. From the limited data available, supportive supervision packages, community monitoring and quality improvement/problem-solving approaches show the most promise; however, evaluation of all strategies is weak. Some strategies are more appropriate for specific settings: For example, self-assessment may be especially helpful for private providers without the benefit of a larger supervisory structure or for providers who are located remotely and cannot benefit from peer or group supervision. It may not, however, be helpful in settings with overall low quality of care as poor performers may not be willing to critique their performance accurately. Group and peer supervision may be most appropriate in settings where supervisors have long distances to travel and where providers are generally supportive of each other and are clustered together. Some level of problem solving may be appropriate whatever supervision strategy is used but may need to focus on areas that are amenable to change. Community supervision may be of particular relevance to CHWs – currently CHW supervision often mirrors formal health worker supervision in aiming to link the CHW and the health system. This focus has been criticized given that the CHWs’ work environment is the community and it has been suggested that their supervision should be focused around links with the community instead (88). A modified version of a community monitoring intervention is currently being trialed in the inSCALE study with CHWs in Uganda (62).

Some themes related to feasibility and implementation cut across the strategies that we reviewed. Providers and supervisors need to have the means to do their work; thus, poor drug supply, high staff turnover, busy clinics, and lack of supervisor transport reduced the impact of supervision. Keeping supervision strategies and methods simple, objective and structured appeared beneficial. This review demonstrates the potential for supervision to be effective, but there is a striking contrast between what we know about supervision and what happens in practice. For example, in a Zambian CHW program, 50% of CHWs had no supervision (32) and even high-profile initiatives such as the Accelerated Strategy of Child Survival and Development (ASCSD) have reported inadequate supervision with 38% of ASCSD CHWs in Mali having never been supervised and 81% reporting a lack of support (31), while in Malawi, 18–22 people made supervision visits to a given clinic giving inconsistent advice (55). Getting supervision to happen is a challenge and donors and governments need to recognize this and support, fund, and manage supervision to ensure best practice is implemented.

When selecting supervision strategies, it is important for implementers to note that supervisors and supervisees are not blank pages. They will perceive supervision based on previous experience and integrate supervision approaches into their existing values, adopting elements that are consistent with their perspectives and possibly rejecting points that are not (89). For example, some health workers in Tanzania viewed supervision as inherently negative and only for weak workers (11), making it potentially difficult for them to accept new supervision approaches as positive and useful tools. Factors such as supervisor background will also impact how an individual approaches supervision and the likelihood that they adopt an innovation. A qualitative study of prevention of maternal to child supervisors in South Africa found that despite the supervisors having the same job description and training, the supervisor who was previously employed in a clerical role took an administrative focus to her supervision while the supervisor who had a counseling background prioritized providing emotional support (90). Despite the importance of the attitude and skills of the supervisor, little is known about determinants of supervisor performance (89) and health workers are often used to supervising CHWs with little thought about their skill set – this is another area where further research would be beneficial.

This narrative review highlights the need for more high-quality research to better understand ways in which supervision systems can be most effective in meeting the unique challenges of employing CHWs to carry out health-related tasks at the community and/or health system levels. This review also highlights the diversity of supervision approaches that policy makers have to choose between with some evidence that supportive supervision, community monitoring, and quality improvement/problem solving may be effective approaches. Whichever approach policy makers choose, investing in quality over quantity is likely to result in more effective supervision.
